# Development and external validation of a nomogram for identifying patients with thyroid eye disease who screen positive for depressive symptoms: a two-center study

**DOI:** 10.3389/fendo.2026.1942052

**Published:** 2026-10-07

**Authors:** Shengzhi Jiao, Liuzi Wang, Jingwen Wang, Wenle Zhang, Jianing Li, Huanhuan Chen, Bei Wang

**Affiliations:** 1 Nanjing University of Chinese Medicine, Nanjing, Jiangsu, China; 2 Affiliated Hospital of Integrated Traditional Chinese and Western Medicine, Nanjing University of Chinese Medicine, Nanjing, Jiangsu, China; 3 Department of Endocrinology, The First Affiliated Hospital with Nanjing Medical University, Nanjing, Jiangsu, China

**Keywords:** depressive symptom screening, external validation, nomogram, prediction model, thyroid eye disease

## Abstract

**Objective:**

To identify factors independently associated with a positive depressive symptom screen in patients with thyroid eye disease (TED) and to develop and externally validate a nomogram-based model for individualized identification in a second-center cohort as a clinically feasible screening tool for prioritizing depressive symptom screening and informing ongoing follow-up and targeted multidisciplinary management.

**Methods:**

In this two-center observational study, a development cohort (n=218; October 2025–January 2026) and an external validation cohort (n=88; February–March 2026) were enrolled from two tertiary hospitals in Jiangsu Province (total 306 patients). A positive depressive symptom screen was defined as a PHQ-9 total score ≥5. Independent associated factors were selected by univariate and multivariate logistic regression. Five models were developed using the same development data and preprocessing pipeline: logistic regression, LASSO, support vector machine, random forest, and XGBoost. The entire modeling process was internally validated and corrected for optimism using 500 bootstrap resamples, and hyperparameters were optimized using stratified five-fold cross-validation. Discrimination (AUC), decision curve analysis (DCA), and external validation were assessed, and the final locked model was applied to the external cohort without updating.

**Results:**

Overall, 116/306 patients (37.9%) screened positive for depressive symptoms. Age, average monthly household income, stressful life events in the past 6 months, EUGOGO severity grade, standardized GO-QoL-AP score, and TRAb level were independent factors associated with a positive depressive symptom screen. The optimism-corrected AUCs of the five models ranged from 0.736 to 0.790, with no statistically significant paired differences between the corrected AUC of logistic regression and those of the other models. Logistic regression was selected and locked. Its apparent development-cohort AUC was 0.815 and its 500-bootstrap-corrected AUC was 0.776 (95% CI, 0.724-0.840; Brier score, 0.186). In external validation, the AUC was 0.742 (95% CI, 0.631-0.843), the Brier score was 0.195, the calibration slope was 0.981, and the calibration intercept was 0.007. At the locked threshold of 0.312, sensitivity was 0.771 and specificity was 0.547. DCA showed some clinical net benefit across threshold probabilities of 0.10-0.50.

**Conclusions:**

We developed a nomogram for identifying patients with TED who screen positive for depressive symptoms based on age, mean monthly household income, stressful life events during the preceding 6 months, EUGOGO severity grade, standardized GO-QoL-AP score, and TRAb level. In second-center external validation, the model showed moderate discrimination, with calibration slope and intercept estimates close to their ideal values. It may serve as an auxiliary tool for prioritizing depressive symptom screening among patients with TED aged 18–59 years.

## 1 Introduction

Thyroid eye disease (TED), also known as thyroid-associated ophthalmopathy (TAO) or Graves’ ophthalmopathy (GO), is a complex autoimmune disorder closely related to thyroid dysfunction. Its prevalence reaches 25%–40% among patients with Graves’ disease; it is the most common extrathyroidal manifestation of Graves’ disease and ranks first among adult orbital diseases in incidence (1–4). It commonly presents with proptosis, eyelid retraction, ocular surface exposure, periorbital soft-tissue swelling, and diplopia; in severe cases it may progress to dysthyroid optic neuropathy (DON) and even blindness (5, 6). In recent years, treatment strategies for TED have expanded to include intravenous glucocorticoids, immunomodulators, biologics, and insulin-like growth factor-1 receptor (IGF-1R) antagonists (7, 8). Even when inflammatory activity is effectively controlled, some patients retain cosmetic and functional impairments such as proptosis, diplopia, and eyelid retraction, and thereby bear a long-term psychological burden (9). This makes TED a sight-threatening and disfiguring disease that seriously affects patients’ quality of life and mental health (10, 11).

Studies have found that the prevalence of depression in TED patients is as high as 18%–33%, significantly higher than in the general population (12–17). Depression is a common psychiatric disorder that can markedly impair an individual’s family, social, and occupational functioning, with a tendency to recur and a high risk of suicide (18). One study showed that, compared with age- and sex-matched controls, patients with Graves’ disease alone did not have a statistically significant increase in suicide mortality, whereas those with concomitant eye disease had a significantly elevated suicide mortality, and this association persisted after adjusting for prior physical and psychiatric illness (19), suggesting that the psychological burden associated with TED may further affect prognosis and even be life-threatening. Moreover, health-related quality of life in TED patients is significantly lower than in the general population and in Graves’ disease patients without eye disease, and depression is an important predictor of impaired quality of life in TED patients (20–22). Therefore, early identification of factors independently associated with a positive depressive symptom screen in TED patients is of great importance for delivering targeted interventions and improving prognosis.

Previous studies indicate that the occurrence of depression in TED patients may be influenced by multiple factors, including disease severity and activity, sex, age, recent psychosocial stressors, and socioeconomic status; however, findings regarding these factors have been inconsistent across studies (10). Although existing clinical screening scales can identify and assess current depressive symptoms, they do not elucidate the multidimensional factors associated with a positive screen specifically in patients with TED. Most related studies have focused on prevalence estimates and associated-factor analyses, and effective tools that integrate multidimensional information to provide individualized estimates remain lacking. In recent years, prediction models have been increasingly applied to risk assessment and clinical decision support in TED, with positive progress in disease diagnosis and severity prediction (23–26); however, research focusing on models for identifying a positive depressive symptom screen in patients with TED remains relatively scarce. In addition, most comparable prior studies performed only internal cross-validation or same-source dataset splitting, lacking validation in an independent external cohort with a different care setting, which limits the extrapolation and clinical translatability of their results.

Accordingly, this study integrated multidimensional clinical and psychosocial information from patients with TED to identify factors independently associated with a current positive depressive symptom screen and to develop a model that estimates an individual’s probability of screening positive. We then evaluated the model’s discrimination, calibration, and clinical net benefit in an independent second-center cohort. The model was intended to provide an objective basis for prioritizing psychological screening and standardized clinical psychological assessment in patients with TED, help clinicians understand the multidimensional factors associated with a positive depressive symptom screen, and support subsequent assessment, ongoing follow-up, and targeted management.

## 2 Materials and methods

### 2.1 Study design

This was a two-center study comprising a model development cohort and an external validation cohort. The development cohort consisted of TED patients treated at a tertiary hospital in Jiangsu Province from October 2025 to January 2026 and was used for model construction and internal validation. The external validation cohort consisted of patients treated at another tertiary hospital in Jiangsu Province from February to March 2026 and was used solely for external validation of the model.

### 2.2 Participants

A total of 306 TED patients from China were recruited. Inclusion criteria: (1) a diagnosis of TED according to the Chinese Guideline on the Diagnosis and Treatment of Thyroid-Associated Ophthalmopathy (2022) (27), confirmed by a clinician; (2) age 18–59 years; (3) ability to understand the study procedures and willingness to participate voluntarily. Exclusion criteria: (1) severe disease of vital organs such as the liver, kidney, or heart; (2) a history of psychiatric illness; (3) pregnancy or lactation; (4) cognitive impairment or communication difficulties. All participants provided written informed consent before enrollment.

### 2.3 Measurement tools

#### 2.3.1 General information questionnaire

A comprehensive information questionnaire was developed on the basis of a thorough literature review and used to collect data in three domains: (1) sociodemographic information: age, sex, place of residence, average monthly household income, education level, and occupation type; (2) clinical characteristics: time since diagnosis, EUGOGO severity grade, and disease activity; (3) laboratory parameters: FT3, FT4, thyroid-stimulating hormone (TSH), and TSH receptor antibody (TRAb) levels.

#### 2.3.2 Patient health questionnaire-9

The PHQ-9, developed by Kroenke et al. (28), comprises nine items. Each item is scored from 0 to 3, giving a total score ranging from 0 to 27, with higher scores indicating more severe depressive symptoms. According to established grading criteria, scores of 0–4, 5–9, 10–14, 15–19, and 20–27 correspond to none/minimal, mild, moderate, moderately severe, and severe depressive symptoms, respectively. Item 9 assesses death-related thoughts and suicidal or self-harm ideation. An item 9 score ≥1 prompted suspension of the assessment and notification of the attending clinician, who evaluated suicide-related risk features. Participants at imminent risk received continuous observation and emergency psychiatric referral. For participants without imminent risk, clinical assessment guided safety-plan development, mental-health referral, and follow-up arrangements.

#### 2.3.3 Life events scale

Used to assess stressful life events over the past 6 months. This scale was developed by the Chinese scholars Yang Desen et al. (29) in the 1980s on the basis of the Social Readjustment Rating Scale (SRRS), adapted to the Chinese social and cultural context, and has since been widely used in China.

#### 2.3.4 GO-QOL appearance subscale score

The appearance subscale (GO-QOL-AP) of the Graves’ Ophthalmopathy Quality of Life questionnaire (GO-QOL) was used to evaluate subjective appearance-related distress in TED patients (30). This subscale contains eight items and mainly assesses impaired self-image, social avoidance, and psychological distress arising from changes in ocular appearance over the past month. Each item has three response categories scored as follows: ‘yes, severely’=1, ‘yes, somewhat’=2, and ‘no, not at all’=3, yielding a raw score of 8-24. Raw scores were converted to a standardized 0–100 score using the recommended formula: standardized GO-QoL-AP score = [(raw score - 8)/16] × 100. Higher standardized scores indicate better appearance-related quality of life and less subjective appearance-related distress.

#### 2.3.5 Assessment of TED severity and activity

TED severity was assessed according to the grading criteria recommended by the European Group on Graves’ Orbitopathy (EUGOGO) (31). Based on the impact of ocular involvement on daily life and the presence of sight-threatening features, TED was classified as mild, moderate-to-severe, or sight-threatening. Mild TED generally refers to ocular features with only minor impact on daily life that are insufficient to justify immunomodulatory treatment or surgical intervention, and may include mild eyelid retraction (<2 mm), mild soft-tissue involvement, exophthalmos <3 mm above normal for the corresponding race and sex, no or intermittent diplopia, and corneal exposure controllable with lubricants. Moderate-to-severe TED refers to disease that does not meet the criteria for sight-threatening disease but has a clear impact on daily life and may require immunomodulatory treatment or surgery, typically presenting with eyelid retraction ≥2 mm, moderate or severe soft-tissue involvement, exophthalmos ≥3 mm above normal for the corresponding race and sex, or inconstant/constant diplopia. Sight-threatening TED is defined by the presence of dysthyroid optic neuropathy and/or corneal breakdown. Patients with a history of dysthyroid optic neuropathy but no active optic nerve involvement or corneal breakdown at the time of the survey were classified into the moderate-to-severe group.

TED inflammatory activity was assessed using the 7-item clinical activity score (CAS). The CAS comprises seven inflammatory signs: spontaneous retrobulbar pain, pain on attempted up- or down-gaze, eyelid erythema, conjunctival injection, swelling of the caruncle or plica, eyelid swelling, and conjunctival edema (chemosis). Each sign is scored as present (1 point) or absent (0 points), giving a total score ranging from 0 to 7. According to EUGOGO recommendations, a CAS ≥3 defines active TED, and a CAS <3 defines inactive TED.

### 2.4 Definition of the primary outcome

The primary outcome was a positive depressive symptom screen, assessed using the validated PHQ-9 self-report scale. A PHQ-9 total score ≥5 was used as the threshold for a positive depressive symptom screen, identifying patients with mild or greater depressive symptoms.

### 2.5 Data collection

Before the formal survey, all investigators received standardized training. During the survey, researchers explained the study purpose, questionnaire requirements, and relevant precautions to patients and distributed the questionnaires after obtaining informed consent. Questionnaires were administered in a one-on-one interview format; for participants who had difficulty completing the questionnaire independently, researchers explained items one by one and assisted in recording responses to minimize missing data and comprehension bias. After completion, investigators reviewed all questionnaires on site to ensure completeness and accuracy. TED severity and disease activity were confirmed by an endocrinology specialist, and two researchers independently entered the data into a computer database in parallel (double data entry). A total of 340 questionnaires were distributed, of which 306 were valid, for a valid response rate of 90%.

### 2.6 Statistical analysis

All statistical analysis were performed using SPSS 26.0 and Python, with Python code run in the PyCharm 2025.3.3 environment. Continuous variables were first tested for normality; those with a normal distribution were expressed as mean ± standard deviation ( $\bar{\text{x}}$ ± s) and compared between groups using the independent-samples t test, while those with a non-normal distribution were expressed as median and interquartile range [M (P25, P75)] and compared using the Mann–Whitney U test. Categorical variables were expressed as frequency and percentage [n (%)] and compared using the χ² test or Fisher’s exact test. A PHQ-9 score ≥5 was used to define a positive depressive symptom screen and served as the outcome variable. Candidate predictors were selected based on clinical relevance and prior literature, and univariate analysis and multivariate logistic regression were used to identify independently associated factors. LR, LASSO, SVM, RF, and XGBoost models were developed using identical data-preprocessing and variable-selection procedures. Hyperparameters were optimized using stratified cross-validation with grid search, and predicted probabilities were calibrated. Sample size adequacy was assessed using Riley et al.’s method for binary-outcome prediction models (32).With six final-model predictor parameters, an outcome proportion of 37.2%, a target overall shrinkage factor of 0.90, a Cox–Snell R² of 0.212 (estimated from the bootstrap-corrected AUC), and both thresholds set at 0.05, the required sample sizes were 224, 143, and 359 for controlling overfitting, limiting R² optimism, and estimating the outcome proportion, respectively; the overall recommended sample size was therefore 359 patient. The development cohort comprised 218 patients and did not meet this requirement. Given the limited sample size, the entire modeling procedure was internally validated and corrected for optimism using 500 bootstrap resamples. Corrected AUCs were compared pairwise across models, with both unadjusted P values and Holm-adjusted results reported. The final model was selected on the basis of overall performance, locked, and externally validated at the second center. All tests were two-sided, and P<0.05 was considered statistically significant.

## 3 Results

### 3.1 Baseline characteristics and cohort comparison

A total of 218 patients were included in the development cohort and 88in the external validation cohort. The participant flow is shown in **Figure 1**. Standardized GO-QoL-AP score, stressful life events reported over the past 6 months, and EUGOGO severity grade differed significantly between the two cohorts (all P<0.05). These differences reflect population heterogeneity between the external validation cohort and the development cohort and help further test the generalizability of the model across different samples (**Table 1**).

**Figure 1:**
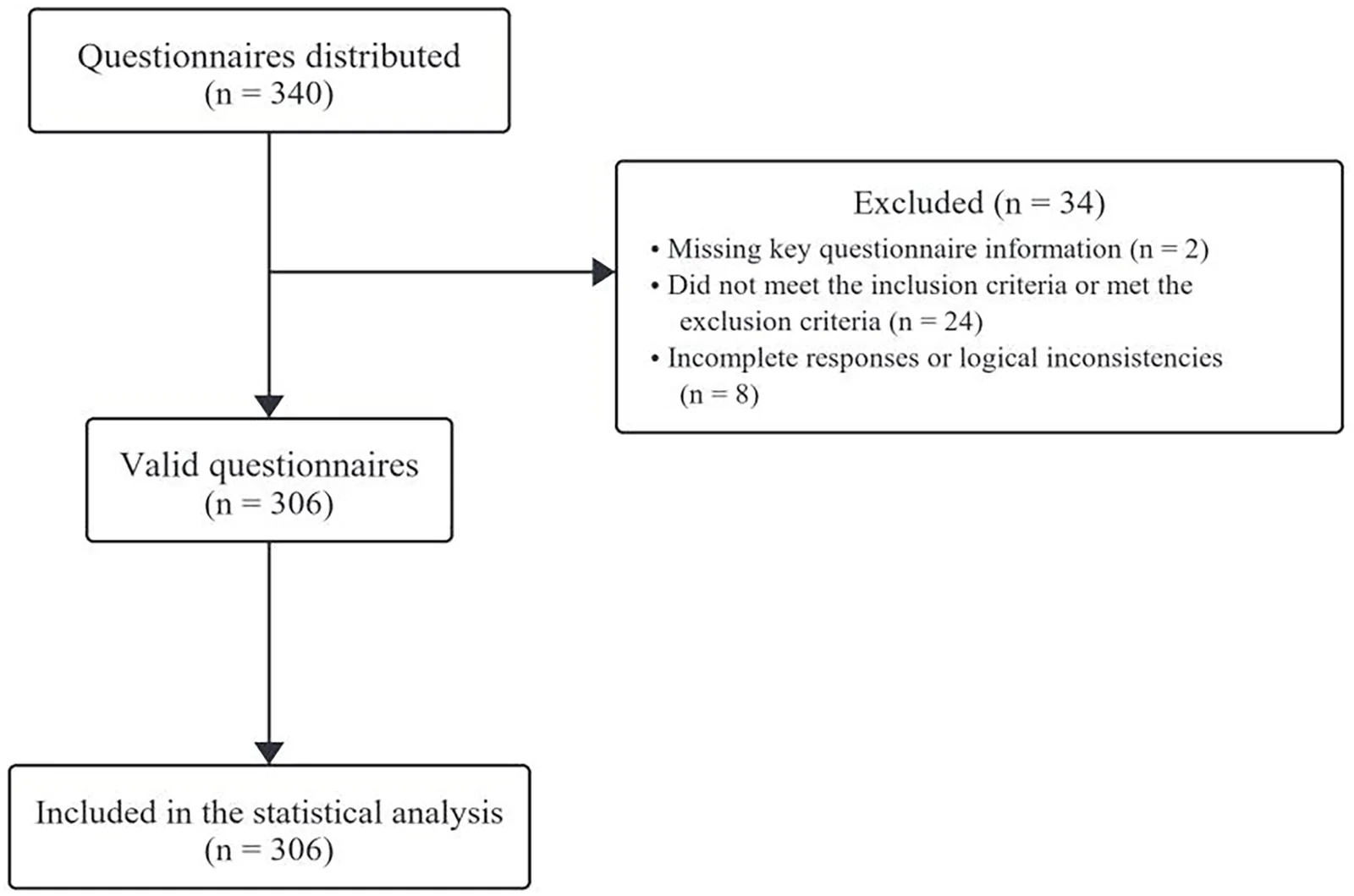
Participant enrollment flowchart.

**Table 1:** Comparison of baseline characteristics between the development and external validation cohorts.

Characteristic	Development cohort (n = 218)	Validation cohort (n = 88)	P value
Age, years	43.50 (34.00, 53.00)	43.00 (32.00, 50.25)	0.504
Sex			0.935
Male	56 (25.7)	23 (26.1)	
Female	162 (74.3)	65 (73.9)	
Place of residence			0.164
Urban	170 (78.0)	62 (70.5)	
Rural	48 (22.0)	26 (29.5)	
Mean monthly household income (RMB)			0.355
<3,000	77 (35.3)	26 (29.5)	
3,000-<5,000	42 (19.3)	23 (26.1)	
5,000-<10,000	61 (28.0)	20 (22.7)	
≥10,000	38 (17.4)	19 (21.6)	
Educational level			0.737
College or above	106 (48.6)	40 (45.5)	
High school/technical secondary school	52 (23.9)	26 (29.5)	
Junior high school or primary school	36 (16.5)	12 (13.6)	
No formal literacy	24 (11.0)	10 (11.4)	
Occupation			0.885
Worker/farmer	35 (16.1)	18 (20.5)	
Civil servant/public-sector official	19 (8.7)	8 (9.1)	
Company employee/technical professional	52 (23.9)	18 (20.5)	
Self-employed	17 (7.8)	9 (10.2)	
Retired	43 (19.7)	15 (17.0)	
Other	52 (23.9)	20 (22.7)	
Time since diagnosis			0.075
≤6 months	103 (47.2)	30 (34.1)	
6–24 months	74 (33.9)	41 (46.6)	
>24 months	41 (18.8)	17 (19.3)	
Active disease (CAS)			0.687
No	144 (66.1)	56 (63.6)	
Yes	74 (33.9)	32 (36.4)	
Stressful life events in the preceding 6 months			0.002
0	119 (54.6)	48 (54.5)	
1	93 (42.7)	29 (33.0)	
≥2	6 (2.8)	11 (12.5)	
EUGOGO severity grade			0.013
Mild	102 (46.8)	55 (62.5)	
Moderate-to-severe (including sight-threatening)	116 (53.2)	33 (37.5)	
Standardized GO-QoL-AP score	81.25 (68.75, 93.75)	68.75 (56.25, 87.50)	0.010
TSH level	1.40 (0.10, 2.84)	1.07 (0.08, 3.23)	0.576
FT3 level	5.00 (4.42, 5.91)	5.04 (4.17, 5.93)	0.725
FT4 level	16.50 (13.43, 19.40)	16.20 (13.10, 18.52)	0.658
TRAb level	2.88 (1.36, 7.26)	3.65 (1.77, 7.86)	0.139
Positive depressive symptom screen			0.669
No	137 (62.8)	53 (60.2)	
Yes	81 (37.2)	35 (39.8)	

All continuous variables showed a skewed distribution on normality testing (D’Agostino–Pearson) and are expressed as median M (P25, P75); between-group comparisons used the Mann–Whitney U test. Categorical variables are expressed as frequency n (%); between-group comparisons used the χ² test, with Fisher’s exact test for 2×2 tables with an expected frequency <5. P<0.05 was considered statistically significant. RMB, Chinese yuan; CAS, clinical activity score; FT3, free triiodothyronine; FT4, free thyroxine; TSH, thyroid-stimulating hormone; TRAb, TSH receptor antibody; GO-QoL-AP, appearance subscale score of the Graves’ Ophthalmopathy Quality of Life questionnaire.

### 3.2 Univariate and multivariate analysis of factors associated with a positive depressive symptom screen in the development cohort

Using a PHQ-9 total score ≥5 as the threshold for a positive depressive symptom screen, patients were divided into a screen-negative group (n=137) and a screen-positive group (n=81). The complete distribution of PHQ-9 scores is shown in **Figure 2**. Because TSH, FT3, FT4, and TRAb levels were markedly right-skewed, they were natural-log transformed (ln) to satisfy the assumption of a linear relationship between continuous predictors and the logit in the subsequent logistic regression, and the transformed lnTSH, lnFT3, lnFT4, and lnTRAb values were entered into all analysis.

**Figure 2:**
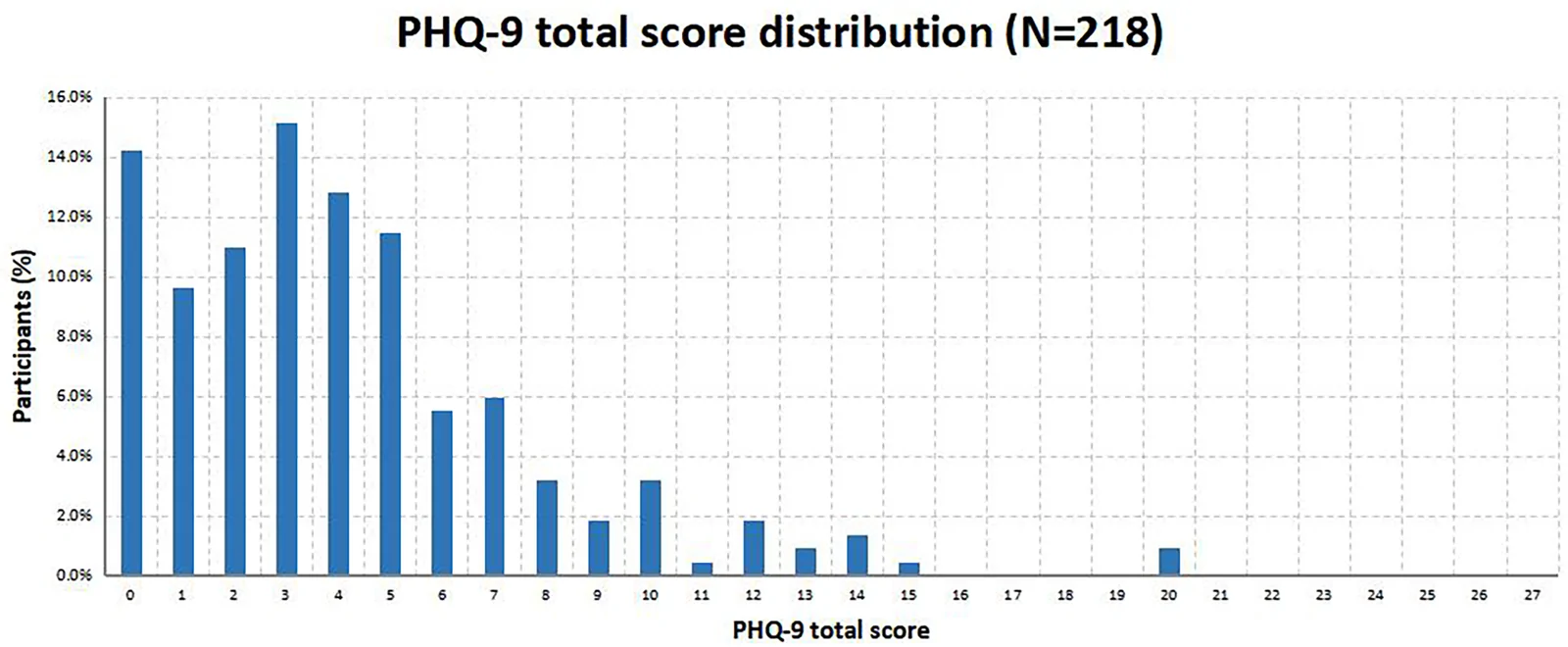
Distribution of PHQ-9 total scores.

Univariate analysis showed that the screen-positive and screen-negative groups differed significantly in age, standardized GO-QoL-AP score, lnTRAb, residence, average monthly household income, stressful life events in the past 6 months, and EUGOGO severity grade (P<0.05), suggesting that these factors may be associated with a positive depressive symptom screen in TED patients (**Table 2**).

**Table 2:** Univariate comparison of predictors between the screen-negative and screen-positive groups in the development cohort.

Characteristic(s)	Negative depressive symptom screen (n = 137)	Positive depressive symptom screen (n = 81)	z/χ^2^	P
Age, years	**44.00 (36.00-55.00)**	**41.00 (31.00-50.00)**	**2.218**	**0.027**
Sex			1.492	0.222
Male	39	17		
Female	98	64		
Place of residence			4.349	0.037
Urban	**113**	**57**		
Rural	**24**	**24**		
Mean monthly household income			12.240	0.007
<3,000	**38**	**39**		
3,000-<5,000	**25**	**17**		
5,000-<10,000	**46**	**15**		
≥10,000	**28**	**10**		
Educational level			2.757	0.431
College or above	68	38		
High school/technical secondary school	36	16		
Junior high school or primary school	19	17		
No formal literacy	14	10		
Occupation			5.865	0.320
Worker/farmer	25	10		
Civil servant/public-sector official	15	4		
Company employee/technical professional	32	20		
Self-employed	9	8		
Retired	28	15		
Other	28	24		
Time since diagnosis			2.720	0.257
≤6 months	68	35		
6–24 months	41	33		
>24 months	28	13		
Active disease (CAS)			0.022	0.881
No	91	53		
Yes	46	28		
Stressful life events in the preceding 6 months			9.975	0.007
0	**86**	**33**		
1	**48**	**45**		
≥2	**3**	**3**		
EUGOGO severity grade			17.516	<0.001
Mild	**79**	**23**		
Moderate-to-severe (including sight-threatening)	**58**	**58**		
Standardized GO-QoL-AP score	**87.50 (75.00-93.75)**	**68.75 (50.00-81.25)**	**6.146**	**<0.001**
lnTSH	0.44 (-1.77-1.10)	0.23 (-3.91-1.02)	1.101	0.271
lnFT3	1.61 (1.49-1.78)	1.60 (1.48-1.75)	0.261	0.794
lnFT4	2.79 (2.57-2.94)	2.84 (2.70-3.01)	-1.733	0.083
lnTRAb	**1.04 (0.14-1.80)**	**1.22 (0.44-2.50)**	**2.094**	**0.036**

Bold indicates statistical significance (P<0.05). Data are median (interquartile range) or n (%). Continuous variables were compared using the Mann–Whitney U test and categorical variables using the χ² test. RMB, Chinese yuan; CAS, clinical activity score; FT3, free triiodothyronine; FT4, free thyroxine; TSH, thyroid-stimulating hormone; TRAb, TSH receptor antibody; GO-QoL-AP, appearance subscale score of the Graves’ Ophthalmopathy Quality of Life questionnaire; PHQ-9, Patient Health Questionnaire-9. Bold indicates statistical significance (P<0.05).

Multivariable logistic regression showed that age, mean monthly household income, stressful life events during the preceding 6 months, EUGOGO severity grade, standardized GO-QoL-AP score, and lnTRAb were independently associated with a positive depressive symptom screen. Age (OR, 0.963; 95% CI, 0.935-0.992; P = 0.014), mean monthly household income (OR, 0.668; 95% CI, 0.487-0.917; P = 0.013), and standardized GO-QoL-AP score (OR, 0.959; 95% CI, 0.941-0.977; P < 0.001) were inversely associated with the outcome. Stressful life events during the preceding 6 months (OR, 2.016; 95% CI, 1.103-3.686; P = 0.023), EUGOGO severity grade (OR, 2.480; 95% CI, 1.225-5.017; P = 0.012), and lnTRAb (OR, 1.325; 95% CI, 1.037-1.692; P = 0.024) were positively associated with the outcome. Place of residence was not significantly associated with a positive screen (OR, 1.980; 95% CI, 0.886-4.423; P = 0.096) (**Table 3**).

**Table 3:** Multivariate analysis of candidate variables associated with a positive depressive symptom screen in the development cohort.

Variable	B	Standard error	Wald χ²	OR (95% CI)	P
**Age, years**	**-0.038**	**0.015**	**6.067**	**0.963 (0.935-0.992)**	**0.014**
Place of residence	0.683	0.410	2.774	1.980 (0.886-4.423)	0.096
**Mean monthly household income**	**-0.403**	**0.162**	**6.216**	**0.668 (0.487-0.917)**	**0.013**
**Stressful life events in the preceding 6 months**	**0.701**	**0.308**	**5.187**	**2.016 (1.103-3.686)**	**0.023**
**EUGOGO severity grade**	**0.908**	**0.360**	**6.377**	**2.480 (1.225-5.017)**	**0.012**
**Standardized GO-QoL-AP score**	**-0.042**	**0.009**	**20.051**	**0.959 (0.941-0.977)**	**<0.001**
**lnTRAb**	**0.281**	**0.125**	**5.068**	**1.325 (1.037-1.692)**	**0.024**

Bold indicates statistical significance (P<0.05). Data are the regression coefficient (B), standard error (SE), Wald χ² value, odds ratio (OR), and its 95% confidence interval (95% CI). RMB, Chinese yuan; LES, Life Events Scale; TRAb, TSH receptor antibody; GO-QoL-AP, appearance subscale score of the Graves’ Ophthalmopathy Quality of Life questionnaire; OR, odds ratio; CI, confidence interval.

A sensitivity analysis was performed using a PHQ-9 total score ≥10 as the threshold for a positive depressive symptom screen. Multivariable logistic regression showed that age, mean monthly household income, stressful life events during the preceding 6 months, EUGOGO severity grade, standardized GO-QoL-AP score, and lnTRAb were independently associated with the outcome. Age (OR, 0.956; 95% CI, 0.920-0.992; P = 0.019), mean monthly household income (OR, 0.516; 95% CI, 0.331-0.804; P = 0.003), and standardized GO-QoL-AP score (OR, 0.960; 95% CI, 0.939-0.981; P < 0.001) were inversely associated with a positive screen. Stressful life events during the preceding 6 months (OR, 2.505; 95% CI, 1.165-5.389; P = 0.019), EUGOGO severity grade (OR, 2.876; 95% CI, 1.082-7.647; P = 0.034), and lnTRAb (OR, 1.712; 95% CI, 1.194-2.453; P = 0.003) were positively associated with a positive screen. The directions of association and statistical conclusions were broadly consistent with the primary analysis. Although fewer positive events widened some 95% CIs, all six variables remained statistically significant, supporting the robustness of the multivariable logistic regression findings (**Table 4**).

**Table 4:** Sensitivity analysis.

Variable	Primary analysis OR (95% CI)	P value	Sensitivity analysis OR (95% CI)	P value	Consistent direction
Age, years	0.963 (0.935-0.992)	0.014	0.956 (0.920-0.992)	0.019	Yes
Place of residence	1.980 (0.886-4.423)	0.096	2.050 (0.766-5.489)	0.153	Yes
Mean monthly household income	0.668 (0.487-0.917)	0.013	0.516 (0.331-0.804)	0.003	Yes
Stressful life events in the preceding 6 months	2.016 (1.103-3.686)	0.023	2.505 (1.165-5.389)	0.019	Yes
EUGOGO severity grade	2.480 (1.225-5.017)	0.012	2.876 (1.082-7.647)	0.034	Yes
Standardized GO-QoL-AP score	0.959 (0.941-0.977)	<0.001	0.960 (0.939-0.981)	<0.001	Yes
lnTRAb	1.325 (1.037-1.692)	0.024	1.712 (1.194-2.453)	0.003	Yes

### 3.3 Model development

Based on the preceding univariate and multivariate logistic regression screening, six variables—age, average monthly household income, stressful life events in the past 6 months, EUGOGO severity grade, standardized GO-QoL-AP score, and lnTRAb—were ultimately included as predictors, and a model for identifying a positive depressive symptom screen (PHQ-9 total score ≥5) was constructed. Variable definitions and coding are shown in **Table 5**.

**Table 5:** Definitions and coding of the outcome and predictor variables used for model development.

Variable	Variable type	Definition and coding
PHQ-9 screening result	Binary outcome	PHQ-9 total score <5 = 0; PHQ-9 total score ≥5 = 1
Age, years	Continuous	Entered as age in completed years, per 1-year increase
Mean monthly household income	Ordinal categorical	0 = <RMB 3,000; 1 = RMB 3,000-<5,000; 2 = RMB 5,000-<10,000; 3 = ≥RMB 10,000
Stressful life events in the preceding 6 months	Ordinal categorical	0 = none; 1 = one event; 2 = ≥2 events
EUGOGO severity grade	Binary	0 = mild; 1 = moderate-to-severe (including sight-threatening)
Standardized GO-QoL-AP score	Continuous	Range, 0-100; higher scores indicate better appearance-related quality of life; entered per 1-point increase
ln(TRAb)	Continuous	Natural logarithm of measured TRAb (IU/L), i.e., ln[TRAb (IU/L)]; entered per 1-unit increase

Mean monthly household income and stressful life events during the preceding 6 months were entered into the model as ordinal numeric variables using the coding shown above. Their regression coefficients represent the change in the log odds of a positive screen for each one-level increase in the coded variable. EUGOGO, European Group on Graves’ Orbitopathy; GO-QoL-AP, Graves’ Ophthalmopathy Quality of Life appearance subscale; TRAb, thyroid-stimulating hormone receptor antibody;PHQ-9, Patient Health Questionnaire-9.TRAb was log-transformed to ln(TRAb) and entered as a continuous variable to preserve full quantitative information and avoid information loss and artificial risk jumps at classification cut-offs.

To avoid bias from any single algorithm and to comprehensively compare different modeling strategies, five machine-learning models—logistic regression, LASSO regression, support vector machine (SVM), random forest (RF), and extreme gradient boosting (XGBoost)—were built using the same development data and preprocessing pipeline. Hyperparameters of LASSO, SVM, RF, and XGBoost were optimized using stratified 5-fold cross-validation combined with grid search (using AUC as the optimization metric), and output probabilities were calibrated using Platt scaling. Missing-data handling, variable selection, standardization, hyperparameter optimization, model fitting, and probability calibration were performed exclusively within the corresponding training data to prevent information leakage.

The complete modeling pipeline for all five models was internally validated and corrected for optimism using 500 bootstrap resamples. In each resample, the process began with the original 15 candidate input variables and repeated missing-data handling, univariable and multivariable selection, standardization, hyperparameter optimization, model fitting, and probability calibration. The six variables selected using the full development cohort represented only the final full-sample fit and were not fixed inputs in the bootstrap resampling process. The same sequence of bootstrap samples was used for all five models. Pairwise differences in optimism-corrected AUCs were calculated for each resample; percentile-based 95% CIs were calculated for the differences, and P values were derived from two-sided empirical tail probabilities. P values for all pairwise comparisons were adjusted for multiple testing using the Holm method. Model selection was based primarily on optimism-corrected AUCs and their paired comparisons. If between-model differences were statistically significant, the model with the best overall discrimination, calibration, and clinical net benefit was to be selected. If the differences were not statistically significant, preference was given to the model with less optimism and better stability, interpretability, and clinical applicability. After selection, the final model was refitted using the complete development cohort, and its preprocessing pipeline, model parameters, probability-calibration method, and decision threshold were locked before direct application to the second-center external validation cohort. External validation results were not used for model selection or parameter updating.

### 3.4 Model validation

In the complete development cohort, apparent AUCs for the five models ranged from 0.810 for LASSO to 0.887 for XGBoost (LR, 0.815; SVM, 0.816; RF, 0.856). Mean optimism ranged from 0.037 for LASSO to 0.098 for XGBoost (LR, 0.040; SVM, 0.080; RF, 0.075). The optimism-corrected AUCs for LR, LASSO, SVM, RF, and XGBoost were 0.776, 0.773, 0.736, 0.782, and 0.790, respectively. Corrected AUCs were lower than apparent AUCs for all models. Mean optimism was relatively small for LR and LASSO but greater for SVM, RF, and XGBoost, suggesting some overfitting in the more complex models. Paired bootstrap comparisons showed that the 95% CIs for differences in corrected AUC between LR and each of LASSO, SVM, RF, and XGBoost all included 0, and all unadjusted P values were >0.05. No other pairwise comparison among the five models was statistically significant, and the conclusions were unchanged after Holm correction (**Figure 3**). Because corrected discrimination did not differ significantly across models, LR was selected as the locked model according to the prespecified selection criteria because it showed relatively little optimism, had a parsimonious structure and directly interpretable coefficients, and was convenient for clinical use (**Figures 3**, **4**).

**Figure 3:**
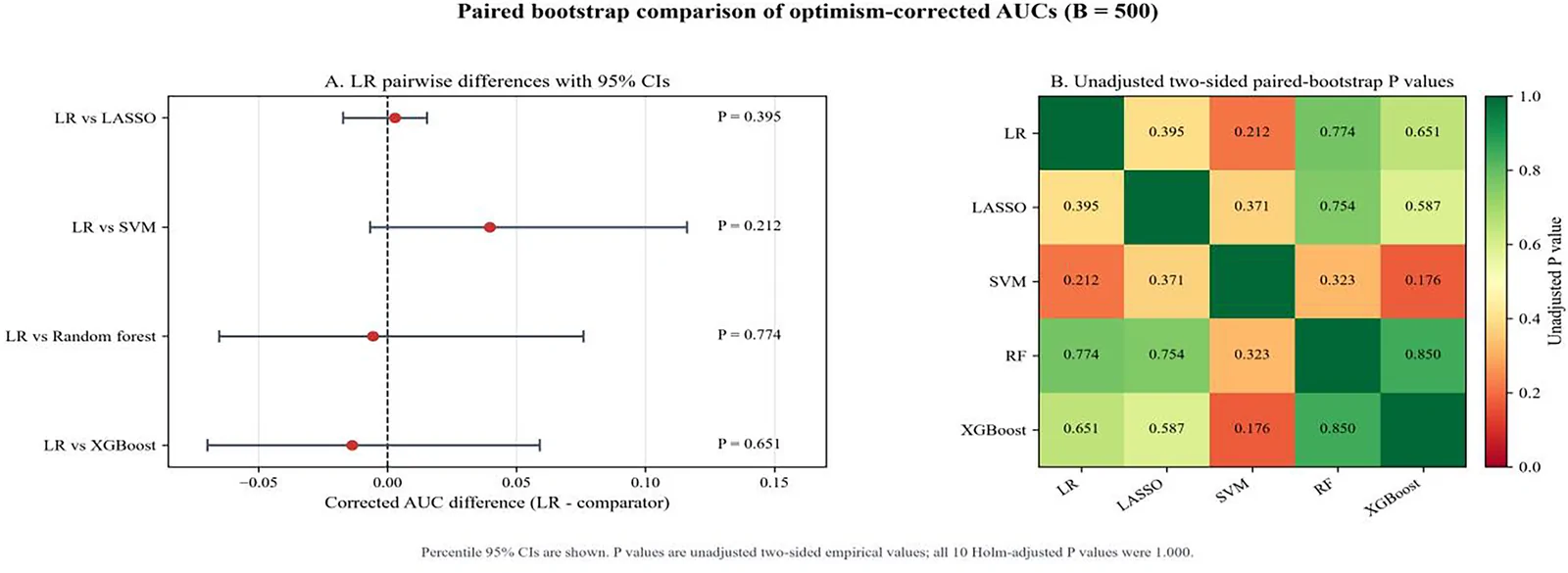
Optimism-corrected AUC differences between LR and comparator models.

**Figure 4:**
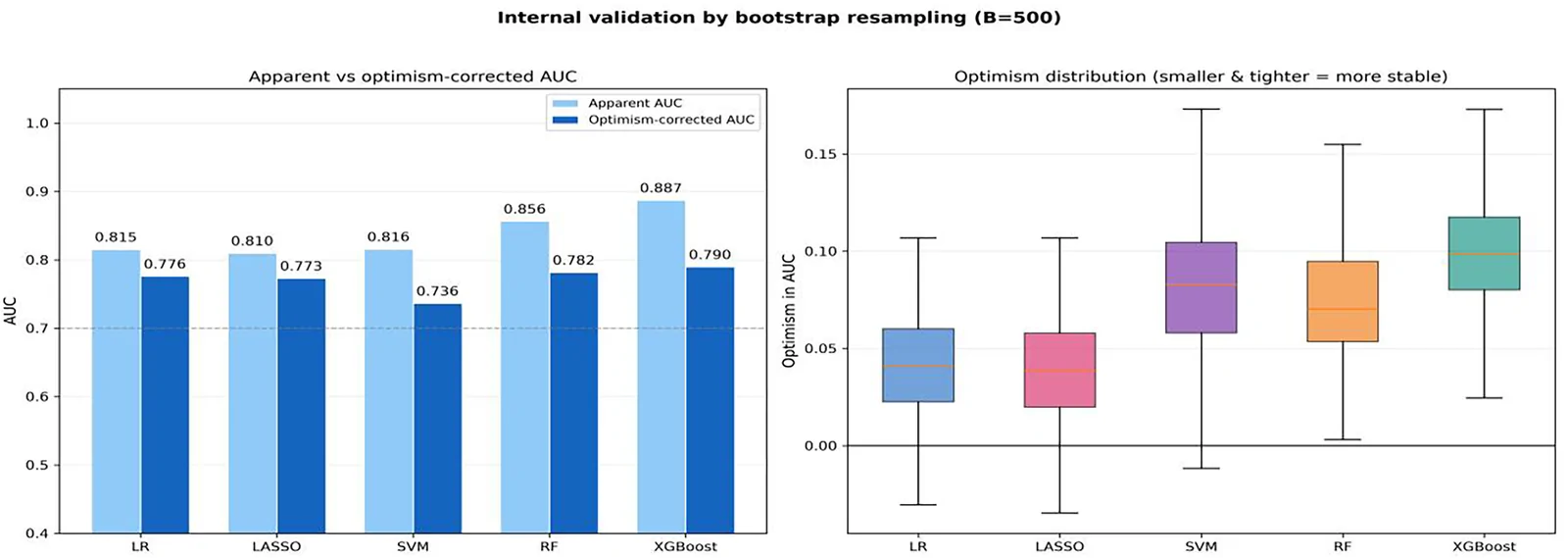
Performance comparison of five prediction models.

### 3.5 Final model and external validation

The final locked model was the LR model. Because no hyperparameter grid was specified for LR, no hyperparameters were selected by grid search. The preprocessing pipeline included median imputation and standardization; imputation values, means, and standard deviations were estimated using only the corresponding training data to prevent information leakage. Neither the development cohort nor the external validation cohort had missing values for the candidate predictors or outcome; consequently, no missing-value imputation was actually performed in this analysis.

On the original variable scale, the linear predictor for the LR model was: LP = 3.7996 - 0.0350 × age - 0.4353 × mean monthly household income - 0.0407 × standardized GO-QoL-AP score + 0.7973 × stressful life events during the preceding 6 months + 0.9264 × EUGOGO severity grade + 0.2441 × ln(TRAb). The value 3.7996 is the intercept of the original logistic regression model. Platt scaling was used to improve agreement between predicted probabilities and the observed proportion of positive screens, with a calibration-fold random seed of 10042. The calibrated predicted probability satisfied the following equation: logit(P) = -0.5729 + 0.6745 × LP. The decision threshold was derived from out-of-fold (OOF) predicted probabilities generated by stratified cross-validation in the development cohort. Among candidate thresholds with sensitivity ≥0.75, the cutoff with the largest Youden index was selected, yielding a final threshold of 0.3123. The global random seed for model development and cross-validation was 42, and the resampling seed for the 500-bootstrap internal validation was 2024. The final model equation, preprocessing pipeline, selected decision threshold, and Platt-calibration relationship were locked after completion of the development-cohort analysis and then applied directly to the external validation cohort.

The final LR model had an apparent AUC of 0.815 in the development cohort and an optimism-corrected AUC of 0.776 (95% CI, 0.724-0.840) after 500 bootstrap resamples, with a corrected Brier score of 0.186, calibration slope of 1.235, and calibration intercept of 0.076. When the locked model was applied without modification to the external validation cohort, the AUC was 0.742 (95% CI, 0.631-0.843), the Brier score was 0.195, the calibration slope was 0.981, and the calibration intercept was 0.007. At the prespecified locked threshold of 0.312, sensitivity was 0.771 (95% CI, 0.599-0.896) and specificity was 0.547 (95% CI, 0.404-0.684). DCA showed that, across the prespecified threshold-probability range of 0.10-0.50, the bootstrap-corrected development-cohort curve and the external-validation curve generally lay above the treat-all and treat-none strategies, indicating some clinical net benefit from using the model to help identify patients who screen positive for depressive symptoms. In external validation, the calibration-intercept and calibration-slope point estimates were close to 0 and 1, respectively, suggesting overall agreement between estimated probabilities and observed outcomes. Overall, the model showed moderate discrimination in the external cohort, with calibration point estimates close to their ideal values; however, the AUC confidence interval was wide and specificity was limited. Its transportability requires further evaluation in larger, independent multicenter cohorts (**Figures 5**, **6**).

**Figure 5:**
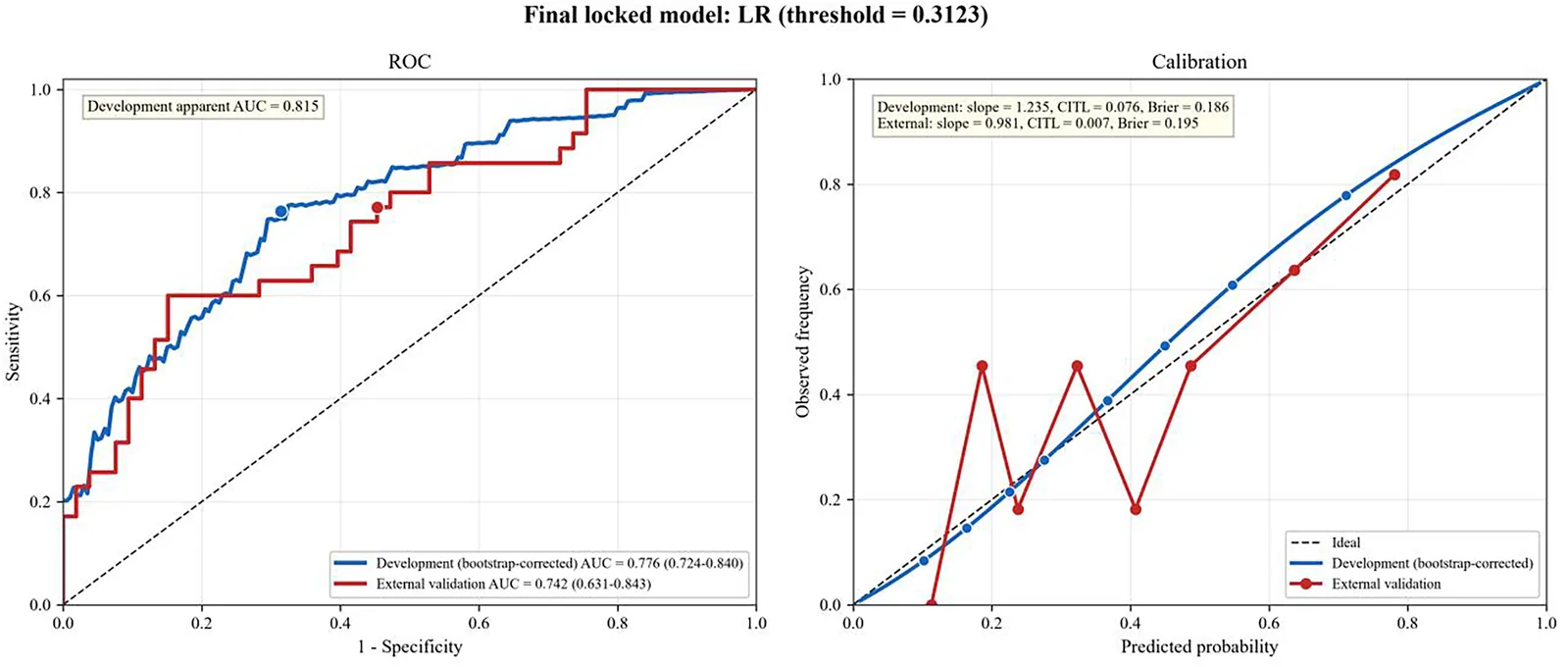
Receiver operating characteristic and calibration curves for the final locked LR model.

**Figure 6:**
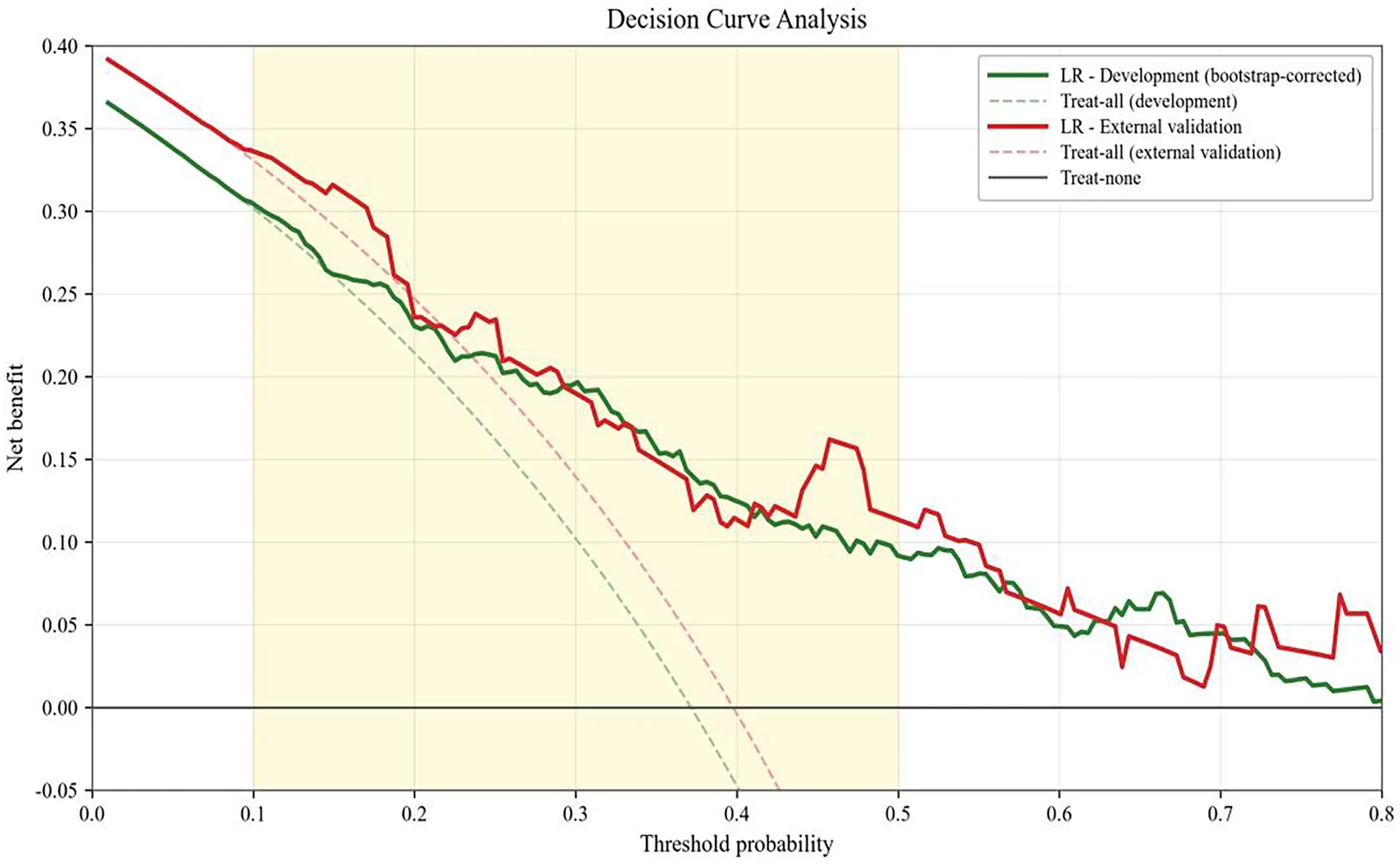
Decision curve analysis of the LR model.

The original development-derived threshold of 0.312 is retained to prioritize screening sensitivity, while thresholds of 0.40 and 0.50 are provided as supplementary operational scenarios for reference evaluation (**Table 6**). Both higher thresholds yielded positive estimated net benefit (0.114 at each threshold), exceeding the assess-all and assess-none strategies at the corresponding thresholds. Compared with the original threshold, 0.40 and 0.50 reduced false-positive classifications by six and 16, respectively, with five and nine additional missed screen-positive cases. The present results show that reducing the assessment burden entails a corresponding trade-off in the risk of missed cases. These supplementary comparisons provide quantitative reference information for different clinical pathways. Greater weight may be given to specificity when a positive model classification directly triggers referral, and to sensitivity when minimizing missed positive screens is the priority.

**Table 6:** Performance of the final locked model at different probability thresholds in the external validation cohort.

Metric	Threshold 0.312	Threshold 0.40	Threshold 0.50
Sensitivity,% (95% CI)	77.1(59.9–89.6)	62.9(44.9–78.5)	51.4(34.0–68.6)
Specificity,% (95% CI)	54.7(40.4–68.4)	66.0(51.7–78.5)	84.9(72.4–93.3)
PPV,% (95% CI)	52.9(38.5–67.1)	55.0(38.5–70.7)	69.2(48.2–85.7)
NPV,%(95%CI)	78.4(61.8–90.2)	72.9(58.2–84.7)	72.6(59.8–83.1)

### 3.6 Model interpretation

Based on the final logistic regression model, a nomogram for identifying a positive depressive symptom screen in TED patients was further constructed. The nomogram incorporates the predictors age, average monthly household income, stressful life events in the past 6 months, EUGOGO grade, standardized GO-QoL-AP score, and TRAb level. For clinical convenience, The nomogram displays TRAb in its original units (IU/L), while point assignments remain based on ln (TRAb) and its original regression coefficient. The total score is obtained by summing the points corresponding to each variable and is then converted into an individualized probability of a current positive depressive symptom screen and used to prioritize screening. According to the prespecified threshold, patients with an estimated probability ≥31.2% were classified as a high-priority group. These patients should receive priority for standardized depressive symptom screening and clinical psychological assessment to evaluate symptom severity, functional impairment, and relevant psychosocial circumstances. Patients below the threshold may receive routine psychological education and longitudinal follow-up based on clinical judgment, together with targeted management addressing TED control, appearance-related and functional impairment, stressful life events, and social support.

For example, a 45-year-old TED patient with a standardized GO-QoL-AP score of 50, a TRAb level of approximately 5.61 IU/L, one stressful life event in the past 6 months, an average monthly household income <3000 RMB, and a moderate-to-severe EUGOGO grade: by mapping each predictor to the points axis and summing them, the patient’s total score is obtained, and mapping the total score to the predicted-probability axis yields an estimated probability of approximately 73% for a positive depressive symptom screen. Because this exceeds the decision threshold, the patient is assigned to the high-priority screening group and should undergo further standardized clinical psychological screening and assessment (**Figure 7**).Manual calculation of ln(TRAb) may pose a practical barrier to bedside application. To facilitate clinical use, we have provided an online calculator at https://sz579-ted.share.connect.posit.cloud/. Users can directly enter the original TRAb concentration (IU/L), and the calculator automatically performs the natural logarithmic transformation and calculates the probability using the original regression equation.

**Figure 7:**
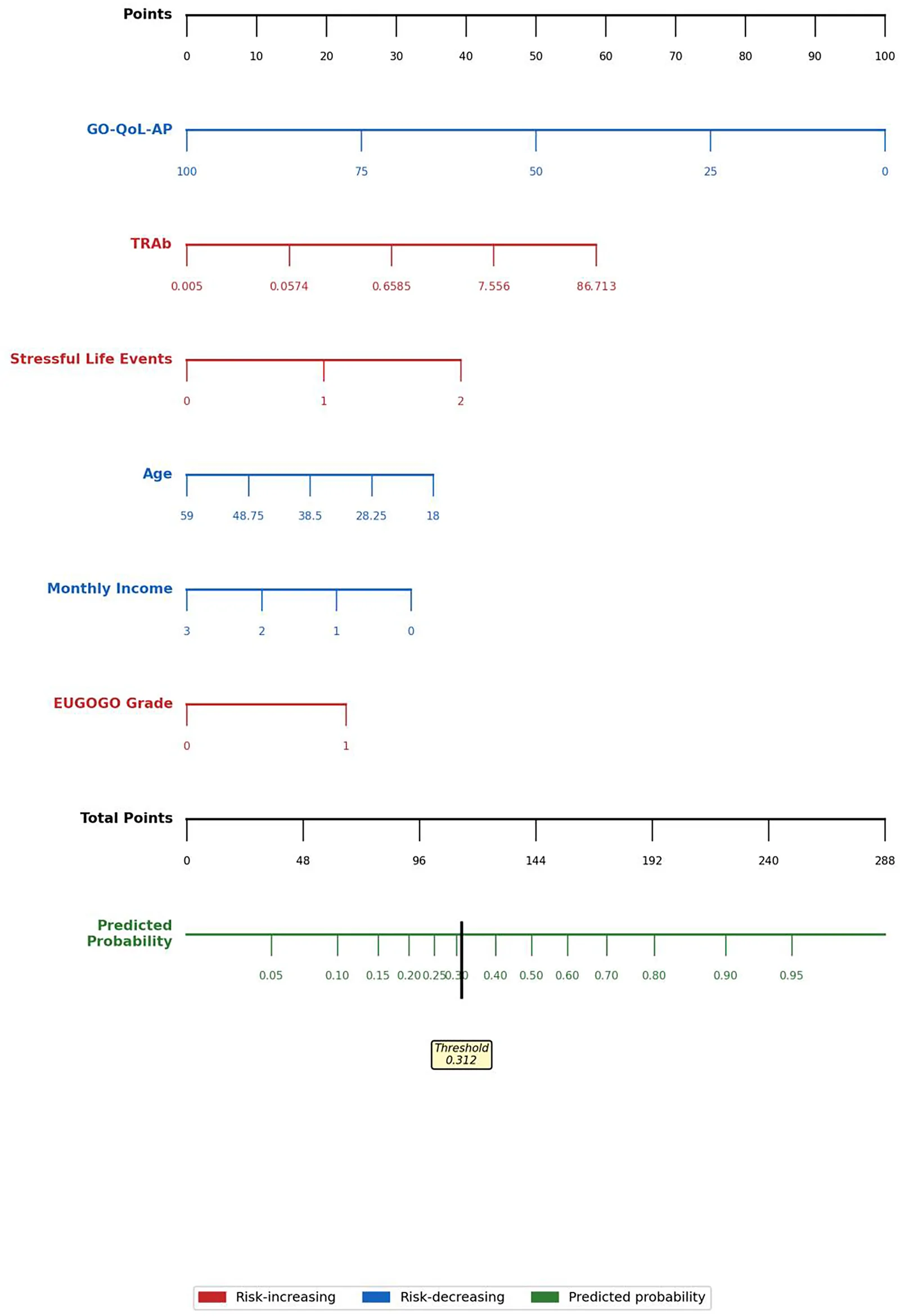
Nomogram for identifying a positive depressive symptom screen in TED patients.

## 4 Discussion

The positive depression-screen rate in this study was 37.9%, indicating that substantial psychological distress is common among TED patients. This rate is broadly close to, but slightly higher than, the 18%-33% reported in previous systematic reviews (12–17), which may be related to our relatively lenient threshold (PHQ-9 total score ≥5) as well as differences in study populations (ethnicity) and assessment tools. This study further identified six factors independently associated with a positive depressive symptom screen—age, average monthly household income, stressful life events in the past 6 months, EUGOGO severity grade, standardized GO-QoL-AP score, and TRAb level—and constructed a model for identifying concurrent screen-positive status.

We found that younger TED patients had a higher likelihood of screening positive for depressive symptoms. Prior research suggests greater psychological vulnerability in younger patients: Zeng et al. (33) found that younger TED patients had lower appearance-subscale scores than older patients, and Holmberg et al. (34) found that younger patients with mild ocular symptoms were more likely to retain psychiatric symptoms 15 months after treatment. Because TED-related changes concentrate in the face—a region of major social significance—and younger patients are often more appearance-sensitive and face higher social-role expectations regarding mate selection, employment, and social interaction, they may be more prone to impaired self-image, social avoidance, and stigma (35, 36). As some studies did not find a significant age–depression association (12, 13), this relationship may be non-linear and influenced by mediating factors such as appearance changes and social expectations, and remains to be verified in larger prospective studies; nonetheless, more proactive psychological assessment of younger patients may be warranted.

TED patients with lower average monthly household income had a relatively higher likelihood of screening positive for depressive symptoms, consistent with research on socioeconomic status (SES) and depression in broader populations. Lorant et al. (37) found that low SES significantly increased depression risk, with a dose-response social gradient, and European multinational studies support low SES as an important predictor (38). Causal research further suggests a bidirectional relationship between poverty and depression/anxiety, whereby economic hardship precipitates depression by increasing chronic stress and eroding coping resources (39). This mechanism may be amplified in TED, which has a long, relapsing course with costly treatments (e.g., glucocorticoid pulse therapy, orbital decompression, strabismus correction); moreover, diplopia and appearance changes can reduce work capacity and income, so that disease and economic burden compound each other (10). Kahaly et al. likewise recorded economic difficulties as a major stressful life event among TED patients (14).

TED patients who experienced stressful life events in the past 6 months had a significantly higher likelihood of screening positive. Studies have shown that 84% of patients experienced a stressful life event within the 6 months before TED onset, positively correlated with depression levels (14). Mechanistically, psychological stress can disturb immune function through the hypothalamic-pituitary-adrenal (HPA) axis and the neuro-endocrine-immune network, inducing or aggravating thyroid autoimmunity in genetically susceptible individuals (40, 41). Stressful life events may act both as a trigger for TED and autoimmunity and as a proximal stressor for depression, making systematic assessment of recent life events and early stress management particularly important for the TED population.

We found that TED patients with a higher EUGOGO severity grade had a greater likelihood of screening positive for depressive symptoms, although prior literature is somewhat controversial. Several studies show that moderate-to-severe patients have significantly higher depression-subscale scores than mild patients (13, 14, 42): a higher grade is usually accompanied by more pronounced proptosis, soft-tissue inflammation, diplopia, and even optic nerve involvement, aggravating psychological stress, and may also reflect a more active inflammatory state, and autoimmune and inflammatory responses are potentially linked to the neuroimmune mechanisms of depression (10, 43). As some studies found no such association (16)—possibly due to heterogeneity in assessment tools, ethnicity, and design—our use of uniform EUGOGO grading and standardized assessment provides supplementary evidence for a positive depressive symptom screen, although prospective verification is still needed.

Greater appearance-related distress (poorer appearance-related quality of life) was associated with a higher likelihood of a positive depressive symptom screen. The GO-QoL captures ‘visual functioning’ and ‘appearance’ (30), and depression is often the main factor impairing GO-QoL (20–22). Wickwar et al. found depressed mood and negative appearance-related cognitions and social anxiety to be important determinants of appearance-related quality of life (12). Female patients report more appearance-related problems (44), and facial/ocular changes are linked to stigma, social avoidance, and depressed mood (34, 35); whether appearance-improving surgery (e.g., orbital decompression, eyelid surgery) also relieves depression warrants further study.

Existing research has not reached a unified conclusion on the association between serum TSH receptor antibody (TRAb) level and depression. Several studies have shown that higher TRAb is associated with increased depression prevalence or severity (45–47). Fukao et al. (47) found that patients with a persistent depressive personality trait had higher TRAb levels and lower remission rates, suggesting a bidirectional influence among psychological stress, depression-related traits, and thyroid autoimmune activity. Neuroimaging offers partial support: Holmberg et al. (48) found smaller amygdala and hippocampal volumes at disease onset—regions involved in emotional processing and stress responses whose structural changes may relate to depression—negatively correlated with TRAb. Song et al. (43) proposed that TRAb-related immune responses can elevate pro-inflammatory cytokines, increase blood-brain barrier permeability, and induce CNS inflammation, while chronic inflammation and HPA-axis activation disrupt monoamine metabolism. However, some studies found no TRAb–depression association, instead implicating subclinical hypothyroidism, Graves’ ophthalmopathy, and elevated TPOAb (49). Thus our finding does not prove causality; TRAb’s effect may also be influenced by TED severity, thyroid status, disease course, and treatment, and its mechanism remains to be elucidated.

We compared LR, LASSO regression, SVM, RF, and XGBoost using the same development cohort and preprocessing pipeline. Optimism-corrected AUCs ranged from 0.736 to 0.790, and paired bootstrap difference tests showed no significant difference between the corrected AUC of LR and those of the other models, indicating no clear performance advantage for the more complex models. According to the prespecified selection criteria, we therefore selected the more parsimonious and interpretable LR model, which is also more convenient for clinical application, and presented it as a nomogram. The final model had a 500-bootstrap-corrected AUC of 0.776 and an external-validation AUC of 0.742, with a calibration slope of 0.981 and intercept of 0.007. Thus, the model showed moderate discrimination, and the external calibration point estimates were close to their ideal values. DCA indicated some clinical net benefit across the prespecified threshold-probability range of 0.10-0.50. By integrating demographic, socioeconomic, psychosocial, and disease-related indicators, the nomogram estimates the individualized probability of a positive depressive symptom screen among patients with TED aged 18–59 years and can help prioritize psychological screening. Compared with use of a depressive symptom scale alone, the model not only estimates a patient’s current probability of screening positive but also highlights factors that warrant attention during subsequent assessment. It may therefore provide quantitative support for prioritizing standardized clinical psychological assessment and screening and for planning ongoing follow-up and targeted multidisciplinary management. The model is an adjunct to depressive symptom screening and cannot replace clinical diagnosis.

This study also has limitations. First, both hospitals are in Jiangsu Province, with relatively homogeneous participant backgrounds, so applicability to other populations requires further validation. Second, the development cohort (n = 218) fell short of the Riley-recommended sample size (n = 359), suggesting that the model’s parameter estimates and performance estimates may be subject to instability and uncertainty, with relatively wide confidence intervals. Bootstrap optimism correction and independent external validation cannot fully compensate for this limitation. Moreover, the nine patients with sight-threatening TED were combined with the moderate-to-severe group, potentially obscuring between-group differences in depressive symptom burden. These findings should therefore be regarded as preliminary and exploratory, warranting validation in larger, multicenter independent cohorts with model updating or recalibration as needed. Third, patients with a history of psychiatric illness were excluded to reduce the potential effects of psychiatric illness and related treatment on PHQ-9 scores and biochemical measures. This may have introduced selection bias, underestimated the depressive symptom burden, and limited real-world applicability. Fourth, depression was screened by the PHQ-9 rather than a structured clinical interview or psychiatric diagnosis, which may introduce screening-related misclassification. Future studies could develop and validate models using a rigorous clinical diagnosis as the outcome. Finally, psychosocial variables such as social support, personality traits, and prior psychological trauma were not included, nor were smoking status, current treatment, and previous orbital surgery; a more comprehensive assessment could further improve model performance. The threshold selected in this study prioritized screening sensitivity (77.1%), although specificity in external validation was relatively limited (54.7%). At higher operating thresholds, improved specificity was accompanied by reduced sensitivity. We therefore explicitly quantified these trade-offs to clarify the model’s performance limitations and provide a concrete basis for considering potential referral burden and missed positive screens within different clinical pathways.

## 5 Conclusion

The nomogram model constructed in this study, based on age, average monthly household income, stressful life events in the past 6 months, EUGOGO severity grade, standardized GO-QoL-AP score, and TRAb level in TED patients, identifies patients who screen positive for depressive symptoms. The model showed moderate discrimination in external validation, with calibration slope and intercept point estimates close to their ideal values and some clinical net benefit. It may serve as an auxiliary tool for prioritizing psychological screening and further assessment among patients with TED aged 18–59 years. Importantly, the nomogram has not been validated in adults aged ≥60 years, a clinically important and vulnerable TED population. The nomogram should therefore not be applied to older patients until it has been specifically validated in this group. Its transportability, decision threshold, and clinical utility require further evaluation in larger, multicenter, and more representative prospective cohorts.

## Data Availability

The raw data supporting the conclusions of this article will be made available by the authors, without undue reservation.
